# Genetic Variation of *Giardia lamblia* Isolates from Food-handlers in Kashan, Central Iran

**Published:** 2017

**Authors:** Hossein HOOSHYAR, Shahrbanou GHAFARINASAB, Mohsen ARBABI, Mahdi DELAVARI, Sima RASTI

**Affiliations:** Dept. of Parasitology, School of Medicine, Kashan University of Medical Sciences, Kashan, Iran

**Keywords:** *Giardia*, Genetic variation, Food-handlers, PCR-RFLP, Iran

## Abstract

**Background:**

Based on genotyping study of human isolates of *Giardia lamblia*; humans are mainly infected by two assemblages A and B. The present study was carried out to determine the sub-assemblages of *G. lamblia* isolated from food handlers referred to Kashan health centers, central Iran, 2015.

**Methods:**

In this cross-sectional study, 3653 stool samples collected from food-handlers that annually refer to health center for getting a health certification and examined microscopically for *G. lamblia* cyst. Totally, 44 isolates were selected from 47 *Giardia* positive samples. Cysts were partially purified by the sucrose density gradient method. After freeze-thaw cycles, genomic DNA was extracted using QIAamp Stool Mini kit. A single step PCR-RFLP method was used to amplify a 458bp fragment at the glutamate dehydrogenase *(gdh*) locus, restriction enzymes *BspLI* and *RsaI* were used for distinguish between genotypes A and B and their subgroups.

**Results:**

Of 44 isolates, 24(54.5%) were sub-assemblage AII, 9(20.5%) group B including 7(15.9%) BIII and 2(4.6%) BIV sub-assemblage and 11(25%) isolates showed a mixed pattern of AII and B. Sub-assemblage AI was not detected in this study.

**Conclusion:**

The higher rate of sub-assemblage AII demonstrated an anthroponotic origin of the infection so infected food-handlers could directly transmit this protozoan to consumers via contaminated food and water. For finding of pattern of transmission and distribution of *Giardia* assemblages and sub-assemblage, more studies in human and animal population in different regions are necessary.

## Introduction

The genus of *Giardia* is one of the most prevalent intestinal parasitic protozoa that infects human and a wide range of vertebrate animals. Human giardiasis is one of the most common intestinal parasitic diseases that cause variable clinical manifestations from the absence of symptoms to acute diarrhea, abdominal pain, dehydration and weight loss (1, 2). The prevalence of human giardiasis is reported from 0.4%–7.5% in industrial countries to 8%–30% in developing countries (3). Based on the morphology of trophozoite and cyst, six species of *Giardia* have been identified in different animals (1). The only species of *Giardia* found in human and some other mammalian animals including livestock and pets is *G. lamblia* (Synonym: *G. duodenalis*, *G. intestinalis*) (3).

Based on the comparison and polymorphisms of glutamate dehydrogenase (*gdh*), the small-subunit of ribosomal RNA (SSU), and triosephosphate isomerase (*tpi*) genes, *G. duodenalis* must be considered as a species complex, whose members are classified to at least eight distinct genetic groups (A to H) or assemblages (1,4). All these assemblages are morphologically similar and are indistinguishable by light microscopy (5). The assemblages C to H are host specific and restricted to animal hosts, however, assemblages C, D, E, and F reported from human as cases occasionally (5, 6). Genotyping study of human isolates of *G. lamblia* in different regions of the world indicated that humans are mainly infected by two assemblages A and B (1, 4, 6–8). Both A and B genetic groups can be considered as zoonotic because these assemblages are found in many species of mammalian animals.

Assemblage A has been divided into four subgroups or clusters (sub-assemblages) AI, AII, AIII, and AIV. Human isolates belong to sub-assemblages AI and AII, while the sub-assemblages of animals are AI, AIII, and AIV. Therefore, only sub-assemblage AI has zoonotic potential and AII is specific for human. For assemblage B, also four sub-assemblages BI, BII, BIII and BIV have been reported. According to available data sub-assemblages, BIII and BIV appear to be found in human, while BI and BII appear to be specific for animals. The BIII sub-assemblage is closer to sub-assemblages BI and BII and therefore has zoonotic potential (6, 9, 10). Using PCR-RFLP to target glutamate dehydrogenase (*gdh*) locus previously has been shown it as a simple, reliable and cost-effective method to identification of assemblages of *G. lamblia* isolated from faces (11–13).

*G. lamblia* is a common intestinal parasite in Iran, especially in rural communities. In different regions of Iran, the reported prevalence of human giardisis varies from 1.2% to 38% (14–16). Despite the high prevalence of giardiasis in some regions of Iran, molecular studies of distribution of human and animal assemblages and sub-assemblages of *Giardia* in Iran are still scarce. A previous study performed in Kerman, south of Iran, from 130 isolates, 60% of samples were found as sub-assemblage AII, 16.7% belonged to sub-assemblage AI and 23.3% were reported as sub-assemblage BIII (17).

Food-handlers are one of the most important sources of distribution and transmission of intestinal parasitic infections including *G. lamblia*, to humans. In Shiraz, Iran 2.6% of cases were infected with *G. lamblia* (18).

No previous studies have been conducted on identification of *G. lamblia* assemblages and sub-assemblages in food-handlers, therefore, this study was carried out in order to determine the sub-assemblages of *G. lamblia* isolated from food handlers referred to Kashan health centers, central Iran, 2015.

## Materials and Methods

### Sample collection

This cross-sectional study was carried out from Mar to Nov 2015 in Kashan, central Iran. Overall, 3653 fecal samples collected from food-handlers that annually refer to health centers for getting a health certification. Demographic data such as age, sex, job and home location (rural or urban) were recorded using a questionnaire.

The study was approved by Ethics Committee of the university.

### Parasitological examination

Stool samples were examined by light microscopy. A Direct wet mount smear for diarrheal samples and a formalin-ethyl acetate concentration method for formed stools samples were performed according to Garcia Protocol in order to parasite diagnosis (19).

*Giardia* positive samples selected for cyst purification. Sucrose gradient with a specific gravity of 0.85 M was used for concentration and purification of cysts from stool materials (20). The cysts washed with distilled water thrice by centrifuging at 600g for 5 min. The pellets were resuspended in 1ml distilled water yielding a concentration of approximately 10^6^ cysts/ml and were stored at −20 °C until further analysis. However, approximately 1 gr of each fresh stool positive samples stored at −20 °C without any preservative solution for direct DNA extraction from fresh positive stool samples.

### DNA extraction

The genomic DNA extraction was carried out from concentrated cyst and some stored fresh positive stool samples using QIAamp DNA Stool Mini Kit (QIAgen Company, Germany) according to the instructions of the manufacturer protocol with some pre-treatment before using kit. Briefly, the sediment containing purified cysts or 0.1 gr of positive faeces is suspended in 200 μL of PBS with 2% PVPP (polyvinylpolypyrrolidone) and stored at −20 °C overnight. After this step, the samples vortexed and followed by 7–10 freeze-thaw cycles in liquid nitrogen and a 100 °C water bath as alternative. The suspension was incubated at 100 °C for 10 min and was used for DNA extraction by QIAamp DNA Stool Mini Kit. All extracted DNA was stored at −20 °C for PCR amplification.

### PCR amplification

A fragment of the glutamate dehydrogenase (*gdh*) gene (458 bp) was amplified by a single PCR using the forward GDHF (5′-TCAACGTCAACCGCGGCTTCCGT-3′) and reverse GDHR (5′-GTTGTCCTTGCACATCTCC-3′) primers as described previously (5,19). DNA amplification was achieved in a total volume of 20 μl. The PCR reaction mixture comprised of (final concentration) 10 mM Tris-HCl (pH=8.9), 50mM KCl, 1.5 mM MgCl_2_, 200 nM each of deoxynucleotide triphosphate (dNTP), 20 *p*mol each of primers and 0.25 mL of Taq DNA polymerase (Takapouzist Co, Iran). Then 1–5 μL of DNA, depending on DNA concentration was added to the reaction mixture and amplified in an automated PCR machine (Flexcycler^2^, Germany).

The PCR conditions were as follows: an initial denaturation step at 94 °C for 5 min and 35 cycles at 94 °C for 45 sec (denaturation), 60 °C for 30 sec (annealing), and 72 °C for 45 sec (extension) with a final extension step for 5 min at 72 °C. A positive sample of *Giardia* DNA and distilled water were used as a negative and positive control. Five μL of each PCR products were separated by electrophoresis on 1.5% agarose gel, stained with ethidium bromide and visualized under ultraviolet light to evaluate success of the reaction.

### Restriction fragment length polymorphism (RFLP)

PCR products were digested with the restriction endonucleases *BspLI* (*Nla IV*) (Fermentas, Lithuania) to distinguish sub-assemblages AI, AII, from assemblages B and *Rsa1* (Fermentas, Lithuania) to distinguish between BIII and BIV sub-assemblages separately.

RFLP analysis was carried out directly on PCR products in a 15 μL reaction volume including 8 μl of PCR product was added to 1X reaction buffer and 1 μL (10 U/ μl) *BspLI* or 1 μL(10 U/ μl ) *Rsa1*. Digestion took place at 37 °C for 3h. The restricted fragments were separated and visualized by electrophoresis on 4% high-resolution grade agarose gel, stained with ethidium bromide and visualized under ultraviolet light. A 50 bp DNA ladder (Yektatajhiz, Iran) was used as a size marker. The assemblages and sub-assemblages were differentiated according to the restriction patterns (Table 1) previously described (5, 12).

**Table 1:** Sizes of expected and diagnostic fragments after digestion of 458 bp PCR product of *Giardia gdh* gen

**Enzyme**	**Fragment**		**Subtype**
	**Expected**	**Diagnostic**	
*BsplI*	16,39,47,87,123,146	87,123,146	AI
	16,39,47,69,77,87,123	69,77,87,123	AII
	47,123,288	47,123,288	B
*RsaI*	30,131,298	131,298	BIII
	30,428	30,428	BIV

## Results

A total of 3653 stool samples were examined for intestinal parasites. Forty-seven (1.28%) individuals were positive for *G. lamblia* cysts. Of 47 individuals infected to *G. lamblia*, 35(74.5%) were male and 12 (25.5%) were female. The mean age was 32.6±13.4.

**Fig. 1:**
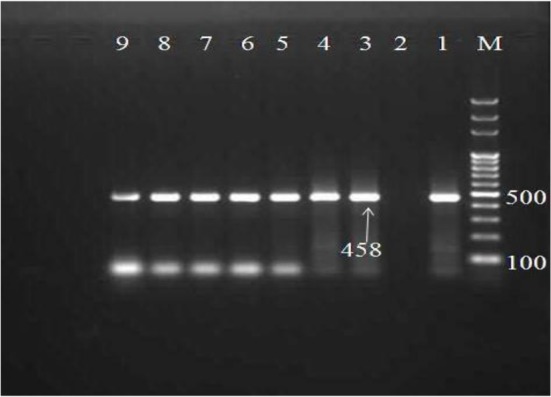
1.5% agarose gel electrophoresis of the PCR product of *Giardia lamblia*, M: 100-bpDNA ladder. Lane 1: Positive control, Lane2: negative, lane3–9 samples

Totally, 44 isolates were selected for DNA extraction. Study of the quantity and quality of extracted DNA showed that QIAamp DNA Stool Mini Kit (QIAgen Company, Germany) was an effective kit for DNA extraction. A 458 bp fragment of *gdh* gene was amplified from all 44 isolates selected for DNA extraction (Fig. 1).

**Fig. 2:**
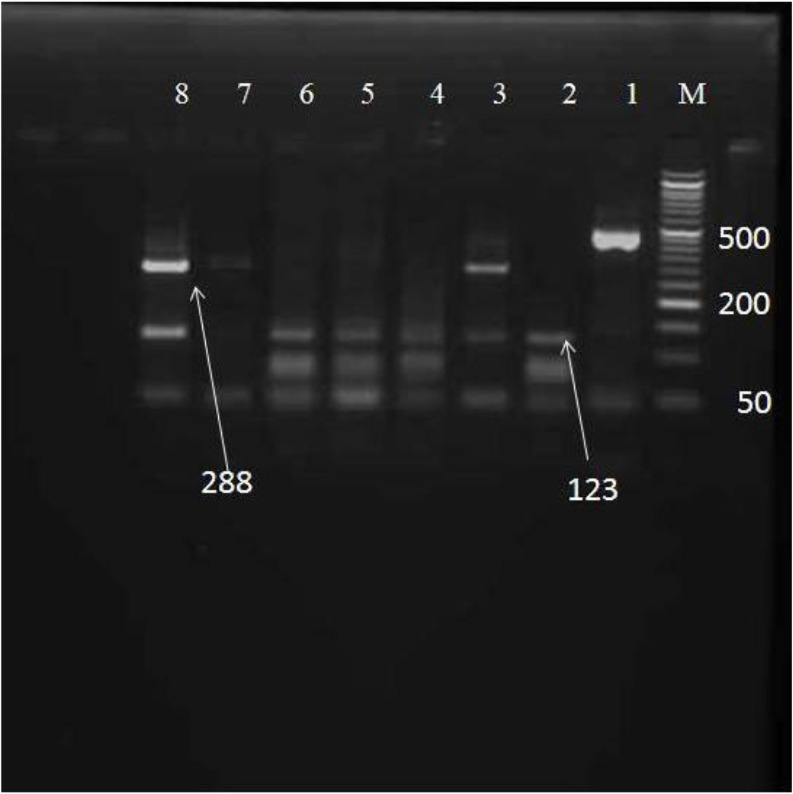
4% agarose gel electrophoresis of the *BsplI* digested PCR product of *Giardia lamblia* M: 50-bp DNA ladder, Lane 1: Undigested product (458 bp), Lane 2, 4–6 assemblage AII, lane 3, 7–8 assemblage B.

PCR products were digested by *BspL1* and *Rsa1* restriction endonucleases, the expected and diagnostic fragments sizes after digestion are listed in Table 1. The digested 458 bp amplified fragment using *BspLI* revealed that 24(54.5%) of the 44 isolates were sub-assemblage AII, 9(20.5%) assemblages B and in 11(25%) isolates shown a mixed pattern of AII and B (Fig. 2).

**Fig. 3:**
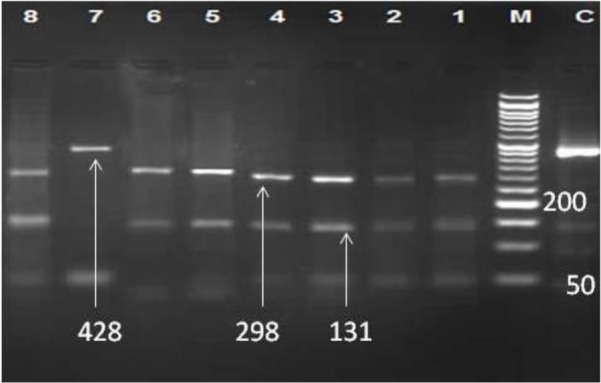
4 percentage agarose gel electrophoresis of the *RsaI* digested PCR product of *Giardia lamblia*. C: Undigested product (458 bp), M: 50-bpDNA ladder, Lane 1–6, 8 assemblage BIII, lane 7 assemblage BIV.

Sub-assemblage AI was not detected in this study. The samples that identified as assemblage B were further processed using the *RsaI* restriction enzyme that revealed 7(15.9%) isolates were sub-assemblage BIII and 2(4.6%) were sub-assemblage BIV (Fig. 3).

## Discussion

According to our results, 54.5% of infected food-handlers had sub-assemblage AII and 25% had mixed infection to AII and B assemblages. Only 4.6% infected to BIII sub-assemblage had zoonotic potential. Sub-assemblage AII is specific for human (6). The higher rate of sub-assemblage AII demonstrated an anthroponotic origin of the infection, so the infected food-handles can directly transmit this protozoan to consumers via contaminated food and water. These persons are important in transmission of *Giardia* since life cycle of *Giardia* is direct and *Giardia* cysts do not need to environmental maturation (1, 21). In this study, a low rate of mixed infection or sub-assemblage BIII shows a zoonotic transmission route as well.

Contaminated food and water are one of most important sources of distribution and transmission of human parasitic diseases. Human can be infected by more than 72 species of protozoan and helminthic parasites through the consumption of contaminated food and water (21). Some of this large number of parasite such as *G. lamblia* shows a cosmopolitan distribution. Food-handlers infected with *G. lamblia* may act as an important carrier for *Giardia* outbreak.

Genotypes of *G. duodenalis* in human fecal sample in different regions and populations in the world showed that differences in the prevalence of assemblages A and B might be attributed to the human population, location, and contact with animals (3, 6, 12, 22). Genotyping of *G. lamblia* in a human population living in a northern Ecuadorian rain forest revealed that from 69 isolates, 42 (61%) were classified as assemblage B (26 as BIII and 16 as BIV), 22 (32%) as assemblage A (3 as AI and 19 as AII) and five (7%) as a mixed AII and BIII types (7). The genotyping of *Giardia* in children was evaluated aged ≤5 yr from Nairobi, Kenya, and 1.4% isolates as assemblage A, 88.9% as assemblage B and 9.7% of mixed infections with assemblages A and B (4).

The present study provides the first data on the assemblages and sub-assemblages of *G. lamblia* in food-handlers in Kashan, central Iran. In our study, the main sub-assemblage of the isolates was AII.

AII as the most common sub-assemblage was followed by BIII and BIV from human in Tehran in central, and Fars province in south of Iran, respectively ( 8,12). Genotyping of human isolate of *G. lamblia* in Isfahan region from a total of 67 isolates, 40 (59.7%) were genotype A sub-assemblage II, 23 (34.32%) genotype B sub-assemblage III and two (2.98%) samples were genotype B sub-assemblage IV. Mixed genotypes of (AII and B) were detected only in two (2.98%) isolates (23).

In contrast, in Ahvaz, southwest of Iran, sub-assemblage BIII was more prevalent among human populations referred to Ahvaz health centers clinics (24). The same results were observed in hospitalized children at Urmia Mutahhari Hospital, West Azerbaijan Province, Iran. 93.3% of clinical samples contained sub-assemblage BIII and 6.7% belonged to the subgroup BIV (25). Sub-assemblage BIII is prevalent in livestock and in these regions; presumably, the infection route was zoonotic origin. For finding the pattern of transmission and distribution of *Giardia* assemblages and sub-assemblages more studies in human and animal population in different regions are necessary. Molecular epidemiological study of pathogenic intestinal protozoa, especially *G. lamblia* will be important for planning the prevention and control programs of these parasites locally and globally.

## Conclusion

54.5% of infected persons had sub-assemblage AII that is specific for human and so food-handlers can be one of the most important sources of transmission of *Giardia* to humans. Some interventions including food-handler’s training, medical check-up and screening parasitological examination of food handlers at least twice in a year, treatment of infected persons and sanitary measurement are necessary for control of *Giardia* transmission.
